# Practices of lactating mothers regarding exclusive breastfeeding in Outapi district, Omusati region: a qualitative study

**DOI:** 10.4314/ahs.v24i4.53

**Published:** 2024-12

**Authors:** Veremunde Nikanor, Emma Maano Nghitanwa, Monika Nakweenda

**Affiliations:** University of Namibia, School of Nursing and Public Health

**Keywords:** Exclusive breastfeeding, practices, lactating mothers

## Abstract

**Introduction:**

Breastfeeding exclusively for the first six months of a child's life is beneficial to the child's health and development.

**Objectives:**

The study objectives were to determine and describe the practices of lactating mothers regarding exclusive breastfeeding and to determine the association between the overall level of practice and demographic characteristics.

**Methods:**

The study employed a quantitative approach which utilised a descriptive- analytic design. The study population was all women in Outapi district breastfeeding babies aged 0 to 6 months. Data was collected from three clinic selected though cluster sampling and 200 participants were sampled through simple random sampling participated in the study. Data were collected in April 2022 using a questionnaire. Data was analysed using a Software Package for Social Science (SPSS VERSION 25.0). Frequency distribution tables and descriptive statistics were generated to summarise data and bivariate analysis was performed to determine the association between sociodemographic characteristics and the level of breastfeeding practices.

**Results:**

The study found that, most 121 (60.5%) participants have poor practice on exclusive breastfeeding. Furthermore, the study found an association between the level of practice and babies' age.

**Conclusion:**

Poor practices regarding exclusive breastfeeding may negatively affect babies' health. Therefore there is a need for reinforcement on the practice as well as health education on the benefits of exclusive breastfeeding. It is recommended that the Ministry of Health and Social Services in Namibia formulate a policy to enforce exclusive breastfeeding among women.

## Introduction

Breastfeeding is an important public health strategy for improving children's and mother's health by reducing child morbidity and mortality and helping to control healthcare costs in the community^1^. The World Health Organization^2^ recommended exclusive breastfeeding for babies from birth up to six months for health and development of the baby. Breastmilk is the healthiest diet for babies because it has a specific nutrient profile of proteins, carbohydrates, and lipids that are essential for proper cell function and growth. Exclusive breastfeeding promotes mothers' health because it prevents the risk of breast cancer and ovarian cysts and it also strengthens the bond between the mothers and their babies^3^. Exclusive breastfeeding means that giving breast milk only to the baby for the first six months in exception of medication, vitamins and minerals^2^. According to the report of the World population data sheet^4^, it has been noted that globally 70% of infants are not exclusively breastfed. Moreover, a study conducted in Ghana found that 79% of the women did not practice exclusive breastfeeding because they have a misunderstanding that breast milk is not enough for the baby^5^. The Ministry of Health and Social Services^6^ indicated that 70% of the babies were not exclusively breastfed in Namibia. Iindongo and Mutorwa^7^, conducted a study in Windhoek, Namibia that found that 70% of lactating mothers have poor practice on exclusive breastfeeding. Omusati Regional annual report of the Ministry of Health and Social Services for 2016/2017 indicated that only 64% of the babies were exclusively breastfed^8^. However, the report for 2017/2018 revealed that the number of breastfed babies decreased to 45%^8^. Despite the decrease in the number of babies exclusively breastfed, the researchers could not find a study conducted regarding practices of lactating mothers on exclusive breastfeeding in Outapi District.

The study objectives were to determine and describe the practices of lactating mothers regarding exclusive breastfeeding and to determine the association between the level of practice and demographic characteristics.

## Methods

A quantitative approach of a descriptive analytical research design was used.

### Setting

The study context was three clinics in Outapi district in Omusati region. Outapi district consist of nine clinics. The selected clinics provides comprehensive primary health care services that include screening, antenatal care, postnatal care and immunization. The Omusati Regional annual report of the Ministry of Health and Social Services for 2017/2018 indicated the decrease in the number of babies exclusively breastfed from 64 % in 2016/2017 to 45% in 2017/2019. Furthermore, it was also stated in the 2017/2018 reports that the percentages of babies who were exclusively breastfed in Outapi district were only 39% which is alarming.

### Population and sampling

The study population was all women breastfeeding babies aged 0 to 6 months in Outapi district during study period. The target population consisted of 401 breastfeeding women attending the clinic with babies aged 0-6 months in nine clinics. Clinics were sampled through cluster whereby three clinics were selected based on attendance such as higher, moderate and low attendance respectively. Simple random sampling was employed to select the participants. A sample size of 200 was calculated using Slovin formula (n =_N/1+N (a)^2^ at a 5% margin of error. The distribution of the sample for each clinic was done according to the proportion of lactating mothers per facility as follows: Outapi clinic 146 (73%), Onawa clinic 39 (19.5%) and Anamulenge clinic 15 (7.5%). The inclusion criteria was all women visited clinics for babies' consultation, either for immunization or treatment with babies aged 0-6 months who are residing in Outapi district. Breast feeding women with babies from 0-6 months from clinics which are not part of the study were excluded.

### Data collection and analysis

Data were collected in April 2022 using interviewer led questionnaire developed by the researchers in English. The data collection tool consisted of two sections that collected data on socio-demographic factors and practices of lactating mothers on exclusive breastfeeding. The registered nurses at each clinic assisted the researchers in identifying participants for the study based on the inclusion criteria.

Validity of the data collection tool was ensured by including only questions relevant to the study objectives in the data collection tool. Furthermore the items on the data collection tool were clear and understandable and derived from the literature. Data collection tool was also reviewed by an experts in the field. The data collection tool was piloted to 20 lactating mothers for reliability. Data were analysed using the Statistical Package for the Social Sciences (SPSS) version 26. Descriptive univariate analysis was performed for each variable, generating frequencies and percentages for sociodemographic characteristics and variables on practices regarding exclusive breastfeeding. Cross tabulation and chi-squared test were utilized to derive the association between participants' overall level of knowledge and sociodemographic characteristics. The level of significance was determined by variable with the p value of less than 0.05% at 95% confidence interval.

### Ethical consideration

The ethical clearance certificate was obtained from the University of Namibia Research Ethics Committee SON3/2020. Permission to conduct the study was granted from the Ministry of Health and Social Services and the management of Outapi district hospital.

Principle of respect for persons was ensured by explaining the study purposes to the participants and ensured that consent form was signed to confirm their willingness to participate in the study. Participants were informed that their participation was voluntary and they can withdraw anytime they wish.

Principle of beneficence was applied as the researchers explained to the participants that they may not directly benefit from the outcome of the research but the findings may be used by the Ministry of Health and Social Services (MOHSS) in exclusive breastfeeding.

The current study did not pose any direct risk or harm as the questionnaires did not have a question that may cause harm to the respondents. However, arrangement was done with social worker at Outapi district for referral in case of need. In addition, participants were selected with the reason related to the research problem and not because they were ready and available for the study. Further, all participants were given the same questionnaire to ensure equal treatment.

## Results

### Sociodemographic characteristics

The mean age of participants was 27.5 years with a standard deviation of 8.2 years. As displayed in table 1 most respondents 83 (41.5%) were in the age category of 21 to 30 years while 16 (8%) were aged 41 years and above years. Most babies of participants, 58 (29%) were 2 months of age. The mean age of babies was 2.6 months with a standard deviation of 1.5 months

**Table 1:** Practices on the time a woman stops breastfeeding exclusively

When to stop breastfeeding exclusively	Frequency	Percentage (%)
3 months	2	1
4 months	17	8.5
5 months	10	5
6 months	116	58
7 months and above	55	27.5
**Total**	**200**	**100**

Most babies were the first babies 56(28%) and the least were the seventh babies which is 7(0.5%). Majority 185(92.5%), of the participants were Wambos. Further, most 138(69%) of the respondents were single, while the rest were either married or divorced. Majority 123 (61.5%) attended secondary school and most participants 137 (68.5%), were unemployed.

### Practices of lactating mothers regarding exclusive breastfeeding

Participants were assessed on the practice concerning the meals that the mother gives to the baby during the first six months. Most participants, 83 (41.5%) revealed that they give their babies porridge, 27 (13.5%) gives potatoes, 33 (16.5%) gives other foods that are not specified, 8 (4%) indicated they give fresh milk while 49 (24.5%) did not respond to the question.

Participants were requested to indicate their practices concerning the number of times the baby is being breastfed. Half 100 (50%) specified that they breastfeed their babies on demand, 91 (45.5%) indicated more than six times, while 9 (4.5%) detailed that they feed their babies less than 6 times. Participants indicated their practice regarding taking the baby out of the breast after each feed. More than half, 106 (53%) indicated that they take their baby out of the breast after each feed when the baby stops sucking, 80 (40%) stated that when the baby does not want the breast anymore while 14 (7%) stated that they take the baby out of the breast when the baby's abdomen becomes big. Participants also indicated their practice concerning the time a woman stops breastfeeding exclusively right time to start complementary food for the baby, and the results are indicated in Table 1. Most participants, 116 (58%) revealed that the time a woman stops breastfeeding exclusively is at 6 months, contradicting 2 (1%) participants that indicated that women stop breastfeeding exclusively at three months. Moreover, participants were assessed on the practice concerning the food that they left for the baby anytime they are not at home to feed the baby during the first 6 months. Below half 93 (46.5%) revealed that they leave their babies with soft porridge, 19 (9.5) leave expressed breast milk, 71 (35.5) gives omaere or oshikundu while 17 (8.5%) indicated that they give formula milk. Participants were also assessed on their practice regarding what the mother does regarding breastfeeding if the baby falls sick during the first 6 months. The majority of respondent 131 (65.5) continue giving breastmilk only, 36 (18%) stop breast feeding, 25 (12.5%) give breast milk and additional formula milk while 8 (4%) indicated that their babies has never been sick during the six months of life.

### Association between the overall level of practice and demographic characteristics

Cross tabulation and the chi-squared test were utilized to derive the association between participants' overall level of practice and demographic characteristics as displayed in table 2. The results indicated a significant association between the overall level of practice and baby's age (p=0.002). However, there is no significant association found between the overall level of practice and health facility (p=0.458), mother age category (p=0.851), birth order (p=0.543), marital status, (p=0.108), education status (p=0.211) employment status, (p=0.643) and ethnicity (p=0.094).

**Table 2:** Association between the overall level of practice and demographic characteristics

Participants' characteristics		The overall level of practice		
		Poor practice	Good practice	P-Value
Health Facility	Outapi Clinic	85 (58.2%)	61 (41.8%)	0.458
	Onawa Clinic	25 (64.1%)	14(35.9%)	
	Anamulenge Clinic	11(73.3%)	4(26.7%)	
Mothers age category	20 years and below	31(62.0%)	19(38.0%)	0.851
	21 years to 30 years	48 (57.8%)	35(42.2.7%)	
	31 to 40 years	33(64.7%)	18 (35.3%)	
	41 years and above	9(56.2.8%)	7 (43.8%)	
Baby's age in months	1	30 (61.2%)	19 (38.8%)	0.002
	2	39 (67.2%)	19(32.3%)	
	3	19(39.6%)	29(60.4%)	
	4	17(94.4%)	1(5.6%)	
	5	4(66.7%)	2(33.3%)	
	6	12(57.1%)	9(42.9%)	
Birth Order	1	31(55.4%)	25(44.6%)	0.543
	2	29 (61.7%)	18 (38.3%)	
	3	23(59.0%)	16(41.0%)	
	4	17(58.6%)	12(41.1%)	
	5	9(69.2%)	4(30.8%)	
	6	3(75.0%)	1(25.0%)	
	7	0(0%)	1(100%)	
	8	6(100%)	0(0%)	
	9	3(60.0%)	2(40.0%)	
Ethnicity	Wambo	108(58.4%)	77(41.6%)	0.094
	Dhemba	12(85.7%)	2(14.3%)	
	Other	1(100%)	0 (0%)	
Marital Status	Single	82 (59.4%)	56 (40.6%)	0.108
	Married	23(69.7%)	10(30.3%)	
	Window	5(100%)	0(0%)	
	Divorced	0(0%)	1(100%)	
	Co-habitating	11(47.8%)	79(52.2%)	
Educational Status	None	17 (48.6%)	18(51.4%)	0.211
	Primary	25(73.5%)	9(26.5%)	
	Secondary	74(60.2%)	49(39.8%)	
	College /University	5(62.5%)	79(39.5%)	
Employment status	Part-time job	9 (64.3%)	3(35.7%)	0.643
	Full-time job	15(55.6%)	12(44.4%)	
	Self-employed	11(50.0%)	11(50.0%)	
	Unemployed	86(62.8%)	51(37.2%)	

### Level of practice on exclusive breastfeeding

All statements under practice regarding exclusive breastfeeding were scored and summed up, and the total was categorized as a poor or good level of practice. The minimum score was 7 and the maximum score was 12. Scores between 7-9 were categorized as poor practice while those between 10-12 were categorized as good practice of exclusive breast feeding. As shown in table 3, majority of the respondents, 121 (60.5%) showed poor practice regarding exclusive breast feeding.

**Table 3:** Level of practice on exclusive breastfeeding

Level of practice on exclusive breastfeeding		
	Frequency	Percentage (%)
Poor practice	121	60.5
Good practice	79	39.5
**Total**	**200**	**100**

## Discussion

The current study revealed that the majority of the participants were aged between 21 to 30 years, and most had babies aged 2 months and were their first babies. The proportion of babies exclusively breastfed varied depending on the age of the baby^7^. Furthermore, the study indicated that 35% of babies aged 0 months, 25% of babies aged 1-2 months were exclusively breastfed, while babies from 3 months were given additional food. The study finding implies that babies aged three months and older ae likely not to be exclusively breastfed.

The current study established that majority of the respondents attended up to secondary school, and they were unemployed. Employment status of the breast feeding mothers may influence their practice of exclusive breastfeeding negatively as employed mothers are less likely to practise exclusive breastfeeding due to the most time spend at work^9^. Additionally, most employed mothers are not exclusively breastfeeding since they can afford to buy formula milk that the infant can be fed on while they are at work. In contrasts, mother's educational level and age at birth have been identified to influence the onset, duration and level of child nutrition with 63 percent of elementary graduate mothers more likely to practice exclusive breastfeeding^10^. Similarly, Saghar, Asghar and Faroog^11^ study found that 95% mothers had information regarding exclusive breastfeeding, however, only half were practising it. Therefore, having knowledge in exclusive breastfeeding does not necessarily translate in to practising it.

The findings of the current study revealed that the baby can be fed with other meals apart from breast milk during the first six months. However, babies who are not exclusively breastfed during the first six months may develop sores in their gut and they may be prone to diseases. The World Health Organisation^2^ defined exclusive breastfeeding as giving a baby only breast milk with no additional foods or liquid with exception of medicines or vitamins if prescribed for the first six months of life. The study also revealed that the mothers give their babes soft porridge and other food during the first six months that indicated poor practices regarding exclusive breastfeeding. Similarly, a study in China found that only 28% of infants were exclusively breastfed because most women believed that breast milk is not enough for the baby, hence they gave additional food to satisfy the babies^12^. This is not in agreement with the WHO^2^ guidelines on infant and young children feeding which recommends that every lactating mother should exclusively breastfeed because breast milk provides complete nutrition for the babies for the first six months. The study further established that most babies were breastfed more than six times a day.

However, the decrease in exclusive breastfeeding and not breastfeeding on demand can be the result of a lack of knowledge of lactating mothers, which may lead to poor practices on exclusive breastfeeding. According to the results of the current study, most participants indicated that they take their babies out of the breast after each feed when the baby stops sucking. In addition, most of the participants revealed that women stop breastfeeding exclusively at six months. This finding is supported by the World Health Organisation^2^ which recommends that mothers should exclusively breastfeed their babies within the first six months of life and partial breastfeeding up to two years of age or older. Moreover, the study revealed that most of the respondents leave their babies with soft porridge to be fed when they are not at home. Thus, Saghar, Asghar and Farooq^11^ revealed that only 49.5% of the mothers fed babies with breast milk only whereas other 17.1% and 10.5% gave breast milk with formula and cow milk respectively up to 6 months. Furthermore, the findings of the current study have shown that the mothers continue giving breast milk even when the baby falls sick during the first 6 months. However, the findings contradicts Outapi District Health Information System8 report that states that only 39% of the babies were exclusively breastfed within the first six months of life. The findings of the current study indicated that 60.5% participants displayed poor practice pertaining to exclusive breast feeding. Poor exclusive breastfeeding practices might be related to poor knowledge about exclusive breastfeeding^7^. The results further indicated a significant association between the overall level of practice and baby's age (p=0.002). This implies that exclusive breastfeeding reduces as the baby months increase. Most mothers indicated adding complementary food after 3 months that might lead to infectious diseases and mortality among babies. Therefore, exclusive breastfeeding should be maintained for the first six months of baby life.

## Limitations

The study used an adequate sample; however the results could not be generalized because it was conducted in one district and one region.

## Conclusion

The findings of this study have shown poor practice among women on exclusive breastfeeding. The study also identified the association between exclusive breastfeeding and baby's age.

What is already know on this topic

- Number of babies exclusively breastfeed at Omusati region and Outapi district decreased.
- Babies not exclusively breastfeed are at risk of suffering from diseases and at risk of higher mortality rate.

What this study adds

- Poor practices on women regarding exclusive breastfeeding identified.

## Figures and Tables

**Figure 1:**
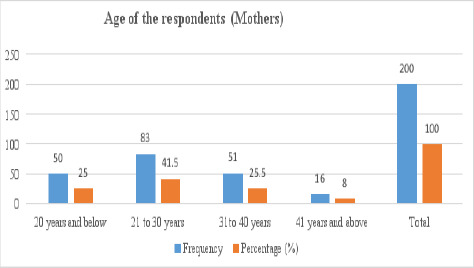
Age of the participants
